# Editorial: Adaptive evolution of plants in mountainous regions

**DOI:** 10.3389/fpls.2023.1296987

**Published:** 2023-10-26

**Authors:** Qian Yang, Ana I.F. Ribeiro-Barros, Luís Silva, Jian-Li Zhao

**Affiliations:** ^1^ Ministry of Education Key Laboratory for Transboundary Ecosecurity of Southwest China, Yunnan Key Laboratory of Plant Reproductive Adaptation and Evolutionary Ecology and Centre for Invasion Biology, Institute of Biodiversity, School of Ecology and Environmental Science, Yunnan University, Kunming, Yunnan, China; ^2^ Higher Institute of Agronomy, University of Lisbon, Lisbon, Portugal; ^3^ Faculdade de Ciências e Tecnologia, University of the Azores, Ponta Delgada, Portugal

**Keywords:** mountains, plants, adaptive evolution, ecological adaptations, climate change, elevational gradients

Mountainous regions hold significant importance in global research on biodiversity and adaptive evolution due to their unique geographical and climatic conditions, as well as the diverse ecosystems they encompass. The variability of elevation and pronounced climatic gradients within mountainous regions can lead to the presence of numerous climatic zones (Wang et al., 2008). This phenomenon facilitates the occurrence of adaptive evolution in plants. Simultaneously, geographical isolation limits gene flow between different mountainous regions, resulting in distinctive adaptive traits across different areas derived from genetic evolution (Hoorn et al., 2010). Leveraging modern high-throughput sequencing technologies and omics data analysis, along with significant morphological trait statistics, offers an in-depth understanding of the mechanisms driving plant adaptive evolution within diverse mountainous environments. Such insights provide a robust scientific foundation for the conservation and preservation of mountainous biodiversity.

Given the relatively limited attention to the study of plant adaptive evolution in mountainous regions, this Research Topic has curated a small collection of research papers encompassing various facets. These include the environmental adaptability expressed through morphological traits, the influence of heterogeneous environments on gene expression, and the impact of mountain roads on plant communities.

Using *Aeonium*, a monophyletic genus of 44, almost all endemic to the Canary Islands, dos Santos et al. demonstrate that growth forms dictate adaptation to local environments. Moreover, specific traits exhibit antagonistic responses to similar environments suggesting that growth forms represent particular ecological functions. Regarding leaf traits and their responses to the environment, Xing et al. conduct a study involving 110 species of three plant functional types (PFT) on the eastern Qinghai-Tibetan Plateau, highlighting the regional-scale variation in leaf traits and the relationships among leaf traits, PFT, and environment (notably, mean annual temperature). Additionally, Sun et al. conduct an investigation on five bamboo species at different elevations in Wuyi Mountain, showing that elevation promotes an isometric scaling relationship between morphological and chemical traits. Particularly, the ratio of leaf width to length (W/L) and the content of phosphorus are the main drivers of photosynthetic capacity across different elevations. In terms of gene expression, Ye et al. conduct transcriptome sequencing on late flower bud and early leaf bud stages of *Rhododendron sanguineum* var. *haemaleum* from four different elevational belts in the Gaoligong Mountains of Southwest of China. The authors report that heterogeneity in the environment induced by elevational change is a primary factor influencing gene expression. This suggests that plant species may employ diverse adaptive strategies to cope with environmental pressures. Li et al. analyze the mechanism of taxonomic homogenization triggered by mountain roads, in 978 species from the Qionglai mountain. The study indicates that the homogenization of community species composition results from the adaptive response of functional traits to environmental consistency induced by roads, as well as from the resorting or reassembly caused by environmental filtering.

The editorial team suggests that further research can be conducted in the following themes to comprehensively explore the adaptive evolution of mountainous plants. Firstly, investigating the reproductive characteristics of plants, such as floral morphology and pollen dissemination mechanisms, can shed light on how plants achieve reproductive success in mountainous environments. Secondly, studying the interactions between mountainous plants and other species, such as the microbiota, insects and birds, can elucidate adaptive co-evolution. Thirdly, examining the adaptive capacity of plants to climate change and exploring their evolutionary responses to changing mountainous climates is crucial. Last, but not the least, high-throughput omics technologies, like genomics, transcriptomics, proteomics, metabolomics, or lipidomics, are of paramount importance to unravel the fundamental aspects of plant adaptation. By amalgamating these research directions, a more comprehensive understanding of the mechanisms undermining adaptive evolution of mountainous plants and their responses to intricate environmental changes can be achieved. The convergence of research across these various directions can mutually corroborate and unveil multiple facets of adaptive evolution.

## Author contributions

QY: Writing – original draft. AR: Writing – review & editing. LS: Writing – review & editing. JZ: Writing – original draft, Writing – review & editing.
